# Prevalence and determinants of stunting and anaemia in children aged 6–23 months: A multilevel analysis from rural Ethiopia

**DOI:** 10.1111/mcn.13736

**Published:** 2024-10-08

**Authors:** Habtamu Guja, Mariana Belgiu, Kaleab Baye, Alfred Stein

**Affiliations:** ^1^ Faculty of Geo‐information Science and Earth Observation (ITC) University of Twente Enschede The Netherlands; ^2^ Center for Food Science and Nutrition, College of Natural and Computational Sciences Addis Ababa University Addis Ababa Ethiopia

**Keywords:** agroecology, anaemia, children 6–23 months, Ethiopia, multilevel analysis, stunting

## Abstract

Low‐ and middle‐income countries shoulder the greatest burden of stunting and anaemia in children. This calls for prompt and effective intervention measures, while the contributing factors are not fully understood. This study evaluates determinants spanning from individual‐, household‐ and community levels including agroecology and antinutrients as unique sets of predictors. Primary data were collected from 660 rural households representing the midland (ML), highland, and upper highland (UHL) agroecological zones from northern Ethiopia. The study relates several predictors to stunting and anaemia in children aged 6–23 months. We found 49.1% and 49.7% of children were stunted and anaemic, respectively. Children living in the ML are approximately twice more likely to be stunted adjusted odds ratio (AOR: 1.869; 95% CI: 1.147–3.043) than in the UHL. The risk of stunting increases by 16.3% and 41.9% for every unit increase in phytate‐to‐zinc and phytate‐to‐iron molar ratios, respectively. A 10% increase in mean aggregated crop yield was observed to reduce the likelihood of stunting occurrence by 13.6%. Households lacking non‐farm income‐generating opportunities, travel longer time to access the marketplace and poor health service utilisation were associated with increased risk of stunting. Low diversity of child's diet, age of the child (18–23 months) and mothers at a younger age are significantly associated with stunting. Risk of anaemia in children is high amongst households with unimproved water, sanitation, and hygiene practices, younger age (6–11 months) and mostly occurs amongst boys. Children in the ML had a 55% reduced risk of being anaemic (AOR: 0.446; 95% CI: 0.273–0.728) as compared to the UHL. Therefore, the influence of these factors should be considered to tailor strategies for reducing undernutrition in children of 6–23 months in rural Ethiopia. Interventions should go beyond the administrative boundaries into targeting agroecological variation.

## Key messages

- Prevalence of stunting and anaemia are significantly varied across the different agroecological zones.
- A lower aggregated crop productivity and higher molar ratios of phytate to iron or zinc are associated with occurrence of stunting.
- At the household level, use of health facilities, access to the market, and involvement in non‐farm income lower the risk of stunting, while improved water, sanitation, and hygiene reduces the occurrence of anaemia.
- Stunting is more common in children aged 18–23 months and with a lower dietary diversity, whereas anaemia is more common in children aged 6–11 months and amongst boys.
- Interventions should consider these contributing factors and go beyond the administrative boundaries into targeting community‐level contextual variations.

## Graphical abstract

Half of the studied children aged 6–23 months are found to be stunted and anaemic, posing serious public health concerns in rural Ethiopia. Several factors are associated with stunting and anaemia at community, household, and individual levels. Interventions should therefore take these contributing factors into account.

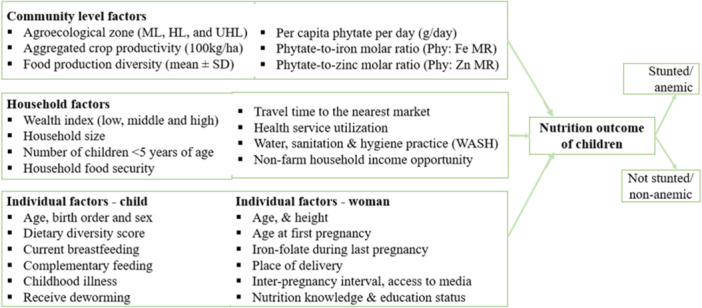

## 1 INTRODUCTION

Stunting and anaemia are composite indicators representing poor child health and nutrition (Dewey & Begum, 2011; WHO, 2021). These conditions are major predictors of impaired cognitive ability and reduced school and work performance. They have been linked to multiple adverse health outcomes that extend to adulthood (Black et al., 2008; WHO, 2023). Low‐ and middle‐income countries shoulder the greatest burden, while children living in rural settings are particularly the most affected (Ekholuenetale et al., 2022; Mishra & Bera, 2024).

Since 2000, global anaemia prevalence in children under 5 has decreased from 48% to 40% in 2019 (WHO, 2021), while stunting has steadily decreased from an estimated 33.0% in 2000 to 22.3% in 2022 (UNICEF/WHO/World Bank, 2023). In developing regions, the progress is slow, with anaemia prevalence remaining at 60% and stunting at 30% in Africa. Even though the trends are pointing downward, the rate is too slow to reach the global nutrition target within 2025 (Weise, 2012) and ending all forms of malnutrition by 2030, as phrased in the Sustainable Development Goal 2 (SDG2; UN, 2015).

To reduce the burden, prompt and effective interventions are thus needed, while in many low‐income countries, including Ethiopia, the contributing factors are not fully understood (Huey & Mehta, 2016). Causes of stunting and anaemia are multifactorial and embedded in the complexity of individual‐, household‐, and community‐level factors. Studies have assessed socioeconomic, demographic, and health‐related factors in relation to stunting (Ayelign & Zerfu, 2021; Berhe et al., 2019) and anaemia (Azmeraw et al., 2023; Endris et al., 2022). Likewise, child age, gender, illness, diet, birth weight and birth order, as well as maternal stature, body mass index, and education were amongst the widely assessed individual‐level factors (Akalu et al., 2021; Berhe et al., 2019; Muche et al., 2021).

While community‐level contextual factors within a country are important determinants in the occurrence of child malnutrition (Ijaiya et al., 2022), large subnational heterogeneity exists in the prevalence of stunting (Amegbor et al., 2020; Uwiringiymana et al., 2022) and anaemia in children (Jember et al., 2021; Locks et al., 2023; Negussie & Nigatu, 2023). This suggests the need for an in‐depth investigation of community‐level factors. Given that rural areas have the greatest share of child undernutrition, several factors including those presumably affecting food production, ultimate utilisation of foods, and nutritional status are of great importance. In this regard, only a few studies have related child nutrition outcomes to environmental factors, like altitude, temperature, or rainfall (Baye & Hirvonen, 2020; Hagos et al., 2014; Randell et al., 2020). Other studies related anthropometric‐based nutrition indicators with agricultural productivity (Amare et al., 2020; Tasic et al., 2020), and food production diversity (Chegere & Stage, 2020; Kumar et al., 2015; Rammohan et al., 2018). Most of these community‐level factors are embedded within a specific agroecological context, while limited studies have assessed the risk of occurrence of child undernutrition across the different agroecological zones.

In Ethiopia, almost two in every five (38%) children under five are stunted, while its prevalence rate reduction is small (CSA and ICF, 2016). Childhood anaemia is a severe public health problem in Ethiopia. The trend amongst children aged 6–59 months shows a decline from 54% in 2005 to 44% in 2011, followed by an increase to 56.6% in 2016 (CSA and ICF, 2016). Given the spatial heterogeneity, interventions to reduce child undernutrition should therefore be geographically targeted, while subnational prevalence may still mask the micro‐level disparities that exist in areas having different agroecological zones within administrative boundaries (Guja et al., 2023).

Agroecological studies benefit from a spatial classification of the landscape with similar agricultural and ecological characteristics (Hurni, 1998). The landscape is then classified by altitudinal ranges with distinct combinations of temperature, rainfall, and length of growing season. In agroecologically diverse countries like Ethiopia, food production and consumption are also likely to vary. Several studies have assessed the role of environmental factors in crop and livestock productivity (Ayalew et al., 2023; Mihretie et al., 2022), examined gross energy and nutrient production (Guja et al., 2023), and have related food production to dietary diversity (DD) at household and individual levels (Baye et al., 2013; Guja et al., 2024; Moges et al., 2022).

Such dietary variables fail to capture seasonal variations in food production due to the temporal mismatch unless the dietary data are collected more frequently (Harris‐Fry et al., 2020). This is due to a shorter reference period of 24‐h or 7‐days for dietary data and a period of 1 year or one season for agricultural production. In this regard, long‐standing nutrition status indicators like stunting and anaemia in children can better represent childcare, diet, and health status over long term.

Both indicators are affected significantly by the deficiency of key micronutrients including iron (Black et al., 2008; Locks et al., 2023; Mutumba et al., 2023) and zinc (Greffeuille et al., 2021; Imdad et al., 2023; Palacios et al., 2020; Umeta et al., 2000; Wastney et al., 2018). Dietary intake of these micronutrients, however, is significantly hampered by the concentration of antinutrients (Gibson et al., 2010; Guja et al., 2023). The problem is intense particularly in low‐income countries where consumption of unfortified cereal‐based complementary feeding is common. Also, the effects of antinutrients on the risk of stunting and anaemia occurrences in children have received little research attention.

The present study aims to relate community‐level determinants including differences in agroecology, production diversity, productivity, and antinutrients with anaemia and stunting in children, in addition to the individual‐ and household‐level factors. From its comprehensive assessment, the study will suggest intervention strategies to reduce anaemia and stunting in children.

## 2 METHODS

### 2.1 Study area

**Figure 1:**
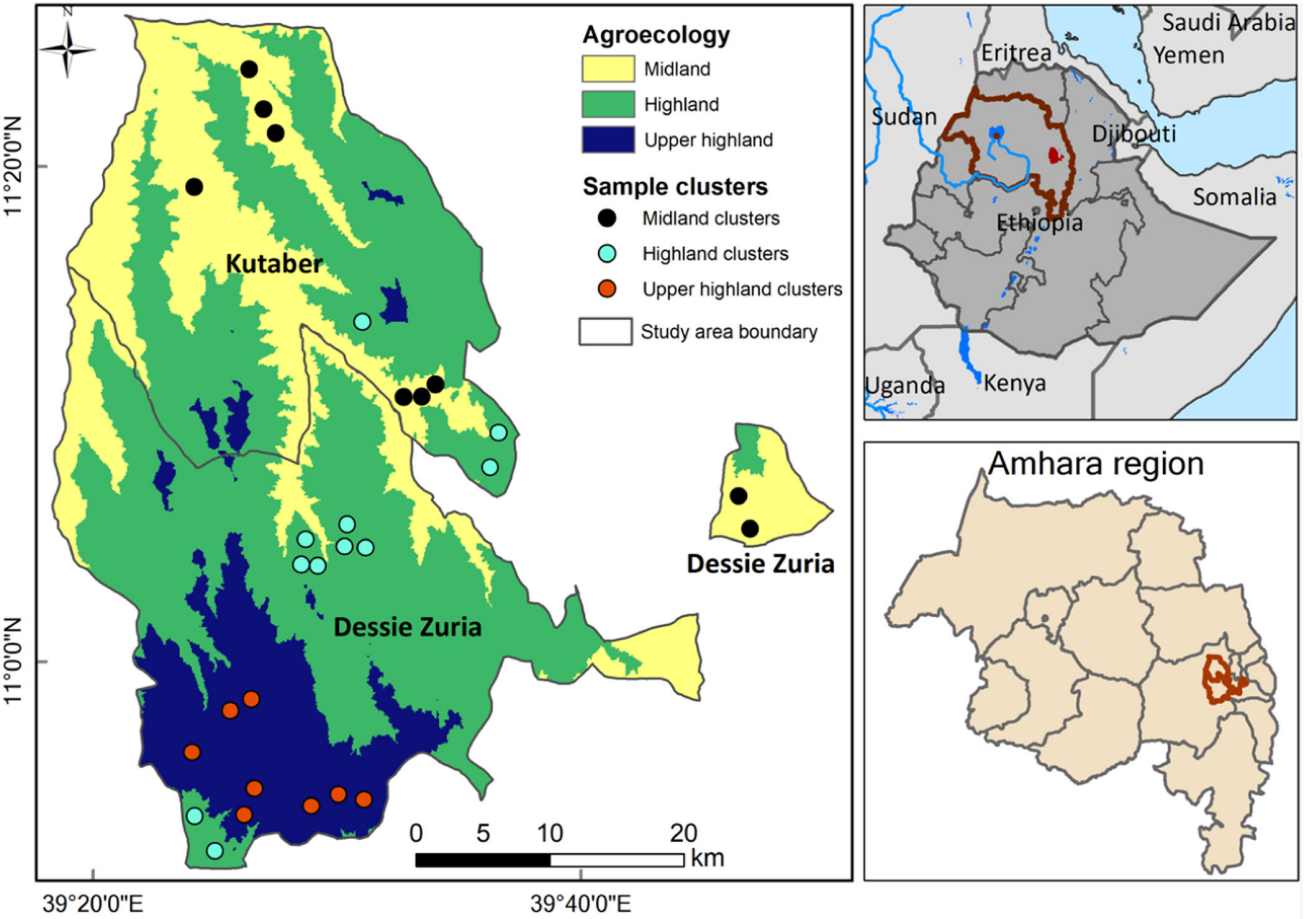
Map of the study area classified by agroecological zones and showing sample clusters in different agroecological zones: Midland (1500–2300 m), highland (2300–3200 m), and upper highlands (3200–3700 m above mean sea level).

Our study was conducted in the Amhara region, located in the northern part of Ethiopia. This region was chosen as it has the greatest burden of stunting, that is, 46% prevalence, which surpasses the national prevalence of 38% (CSA and ICF, 2016), and it has diverse agroecological zones. This area thus provides an opportunity to examine agroecological zones as community‐level determinants in relation to children nutrition outcome. Figure 1 shows the study area representing the three agroecological zones: midland (ML), highland (HL), and upper highland (UHL) within two predominantly rural districts in South Wollo.

### 2.2 Study design and sampling

A community‐based cross‐sectional study design was implemented (Setia, 2016), involving 660 mother‐ or caregiver‐to‐child pairs. A multistage cluster sampling was used to select the study participants. Initially, the study districts were purposefully selected for being predominantly rural and representing various agroecological conditions. Within the districts, villages (*kebeles*) representing the three agroecological zones (AEZs) were chosen using simple random sampling. This study considered various aggregates within the villages/communities to set the number and size of study clusters. This resulted into 28 randomly chosen clusters having an average size of 27 mother‐to‐child or caregiver‐to‐child pairs in each cluster, distributed over the different agroecological zones (Figure 1). Within the chosen clusters, mothers or caregivers with children aged 6–23 months were selected using systematic random sampling. From mothers or caregivers who provided their informed consent, all required household and individual‐level data were collected. Anthropometric and haemoglobin measurements were taken from children aged 6–23 months.

### 2.3 Data collection

Household and individual data were collected from March to April 2021 using a structured questionnaire that was administered by an interviewer. Enumerators received at least 2 days training before data collection, and those responsible for taking anthropometric and biochemical measurements received an additional day of training. Supervisors were assigned to oversee the work of the enumerators and facilitate good rapport with community members. The data collection method for the study variables are indicated below.

#### 2.3.1 Anthropometric measures

Child weight was measured using a calibrated SECA electronic scale to a precision of 0.1 kg and child length was measured in a recumbent position to the nearest 0.1 cm by using a portable board with an upright movable wooden base. Two measurements were taken for all anthropometric measurements, and the average was used for estimating nutritional status (Moss et al., 2020).

Child malnutrition indicators stunting, wasting, and underweight refer to each child: it could be too short for its age (low length‐for‐age); too thin for its length (low weight‐for‐length); and low weight‐for‐age, respectively. Using the WHO Multicentre Growth Reference (2006), children with a length‐for‐age *z*‐score, a weight‐for‐length *z*‐score, and weight for age *z*‐score (WAZ) below –2 are defined as having stunting, wasting, and underweight, respectively. We used the Anthro software of the World Health Organization for the computation of *z*‐score that considers a child's gender, age, length (cm), and weight (kg).

#### 2.3.2 Haemoglobin (Hb) determination

Hb concentration was measured by trained medical laboratory technicians, who performed tests on fingertip blood samples collected from all children. These analyses were performed using the HemoCue Hb 301 haemoglobin analyser (HemoCue Inc, Ängelholm, Sweden). Hb concentration was adjusted both for altitude and method followed for the determination. The partial pressure of oxygen in the atmosphere decreases with increasing altitude and chronic exposure to such environmental hypoxia increases erythropoiesis including Hb concentration as an adaptive response to lower oxygen saturation of the blood (Alarcón‐Yaquetto et al., 2022; Sharma et al., 2019). Owing to these adaptive increases in Hb, the correct interpretation of Hb concentration to identify anaemia requires adjusting for altitude. The following equation was used to adjust Hb for altitude (Alt) (Sharma et al., 2019):

$${\mathrm{Hb}}_{\mathrm{adj}}(g/\mathrm{dL})=-0.032\times \left(\mathrm{Alt}∙0.0032808\right)+0.022\times {\left(\mathrm{Alt}∙0.0032808\right)}^{2}.$$

Comparative studies matched by time but using different methods for determining Hb concentration have shown inconsistencies in estimates of Hb (De la Cruz‐Góngora et al., 2022; Hinnouho et al., 2018; Patel et al., 2013). Therefore, this study considered estimating measurement errors in Hb quantification in HemoCue Hb 301 and compared the results against a reference method in automated haematology analyser (Beckman Coulter Haematology Analyser).

The same samples were measured using a reference method and HemoCue Hb 301. The mean value of the difference differed significantly on the basis of Bland–Altman plot and *t*‐test, showing the presence of bias (Coskun, 2024; Giavarina, 2015). Linear regression then applied to further distinguish whether this systematic bias was fixed or proportional (Ludbrook, 2010). Fixed bias was found, and corrections to the resulting mean difference were done to all readings carried out through HemoCue Hb 301.

A child was categorised as anaemic if the Hb concentration is less than 10.5 g dL^−1^. Based on severity, anaemic children were further classified into three: mild, 9.5–10.4 g dL^−^^1^; moderate, 7‐9.4 g dL^−^^1^; and severe, <7 g dL^−^^1^ (WHO, 2024). Children with moderate to severe anaemia were provided with age‐appropriate iron and multivitamin preparation. These are used as part of intervention at primary healthcare facilities, and then referred to a local health facilities for further evaluation and treatment. This particularly happened in places where the nearest health facility lacks these supplemental preparations and the studied households do not have health insurance.

#### 2.3.3 Factors associated with anaemia and stunting in children

Community‐level estimates of aggregated crop productivity (yield) and antinutrient concentration were determined using the method described in Guja et al. (2023). Community‐level production diversity was obtained by recategorizing production into seven food groups appropriate for child DD (WHO, 2008). The food groups are constructed from crop and livestock species grown or raised by the household in the year, including products from livestock and homestead garden.

Asset‐based indices and housing characteristics were used to construct a relative household wealth index, due to the unavailability of reliable income and expenditure data in rural households. It was obtained from a principal component analysis on household durable assets, land size, livestock owned, housing characteristics, and access to community infrastructures (Henry, 2003).

Utilisation of health service is defined whether a woman had ≥4 antenatal care (ANC) visits during her most recent pregnancy (Headey, 2019; Hirvonen, 2019) or members of the household visited healthcare facility in the previous 3 months before the survey (Dagnaw et al., 2022). Safe drinking water supply, treated water, and improved sanitation facilities were defined according to the UNICEF and WHO's Joint Monitoring Programme (WHO/UNICEF Joint Water Supply, & Sanitation Monitoring Programme, 2015). Household food insecurity status was measured by using the household food insecurity access scale (Coates et al., 2007).

Nutrition knowledge of women was tested using a questionnaire adapted from FAO (Marías & Glasauer, 2014). The questions covered knowledge of food sources related to nutrients, dietary recommendations, and the relationship between diet and diseases. Woman with an average or higher score was considered to have good nutrition knowledge. Inter‐pregnancy interval of ≥24 months between a child's births to the next conception as the safe spacing for both mothers and new‐born baby's health and nutrition status is considered according to the WHO (2007) recommendation.

During their most recent pregnancy, women were asked whether they gave birth at a health facility and took iron‐folate supplement as recommended by the healthcare providers (self‐reported). A child considered to have presented with illness if it appears with any common childhood illness including fever, diarrhoea, cough, or breathing difficulty in the previous 2 weeks before the survey as indicated in Ullah et al. (2019).

### 2.4 Statistical analysis

Data that are sampled in a multistage manner have a structure of hierarchically ordered units (Raudenbush & Bryk, 2002). In this study, we have variables describing individuals (i.e., children), while children are nested within households from communities that are differing in agroecology and food production related variables. Thus, modelling data by ignoring this nesting can lead to false inferences about the relations amongst variables in the model (Lee & Hong, 2021).

**Figure 2:**
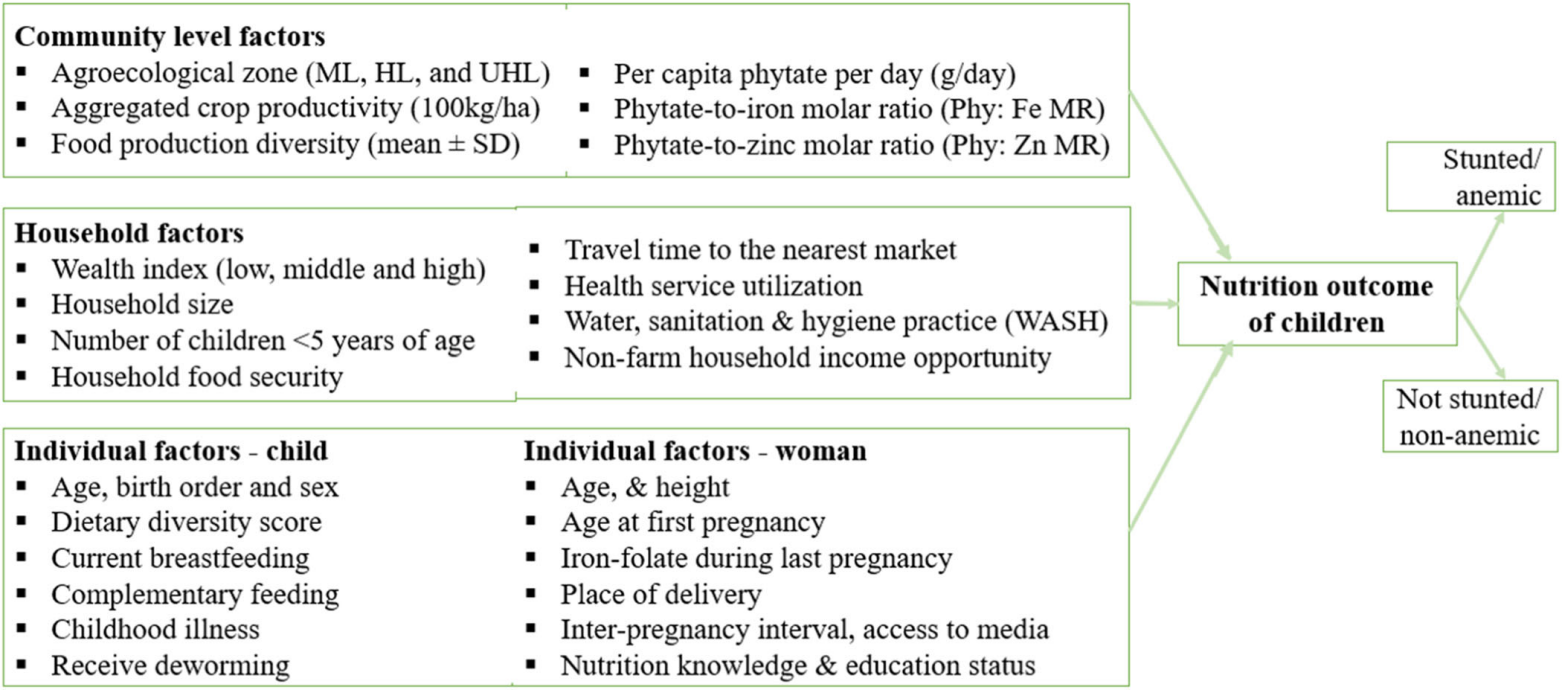
Framework for analysing factors associated with stunting and anaemia amongst children aged 6–23 months from south Wollo, Ethiopia.

A multivariate binary logistic regression model was fitted considering several predictor variables listed hierarchically in Figure 2. The effect of each predictor variable on the outcome variable was checked independently at a significance level of *p* ≤ 0.25 (Stoltzfus, 2011), and those variables that are statistically significant at the bivariate logistic regression analysis were considered as candidates for multivariable analysis. Backward stepwise logistic regression was used to select predictor variables into the final models. We considered those variables that are important for the study's purpose even though they were not included in the stepwise variable selection process (Agresti, 2012). Finally, a multivariable analysis was performed to estimate adjusted odds ratio (AOR) with 95% confidence interval (CI) at a significance level of *p* < 0.05.

In the present study, let $Y_{1i}$ and $Y_{2i}$ be the dichotomic outcome of stunting and anaemia of the *i*th children aged 6–23 months, respectively. For dichotomic outcomes $Y_{\mathrm{ji}},j=1,2$ and a vector of independent variables *X*, multivariate binary logistic regression model is given by (Agresti, 2012):

$$\pi_{j}\left(X\right)=\frac{e^{\beta_{j_{0}}+\beta_{j_{1}}X_{1}+\beta_{j_{2}}X_{2}+\beta_{j_{3}}X_{3}+∙∙∙+\beta_{j_{p}}X_{p}}}{1+e^{\beta_{j_{0}}+\beta_{j_{1}}X_{1}+\beta_{j_{2}}X_{2}+\beta_{j_{3}}X_{3}+∙∙∙+\beta_{j_{p}}X_{p}}}=\frac{e^{\beta_{j}X}}{1+e^{\beta_{j}X}},j=1,\;2$$

where $\pi_{j}\left(X\right)$ = $P\left(Y_{\mathrm{ji}}=1/X\right)$, the probability of the *i*th child aged 6–23 months being stunted ($Y_{1i}$) or anaemic ($Y_{2i}$) given the predictors *X*. The logit (log odds) that marked linear association with independent variables can be stated as:

$$\begin{array}{ccl} logit\left[P\left(Y_{ji}=1/X\right)\right] & = & \beta_{j_{0}}+\beta_{j_{1}}X_{1}+\beta_{j_{2}}X_{2}+\beta_{j_{3}}X_{3}\\ & & +\;∙∙∙+\beta_{j_{p}}X_{p}={X\beta }_{j},\;j=1,\;2 \end{array}$$

Predictor variables $X_{1}$ through $X_{p}$ included individual‐, household‐, and community‐level factors (see Figure 2). In the process, we checked for multicollinearity using variance inflation factor (VIF) and correlation between variables. VIF values <10 and variables with a correlation coefficient of *r* <0.7 were considered to indicate absence of multicollinearity (Dormann et al., 2013; Midi et al., 2010). For all tests, a *p* < 0.05 was considered statistically significant. We report on AOR with a 95% CI and the unadjusted crude odds ratios (COR) was reported only in few cases. Data were analysed by SPSS version 29.

### 2.5 Ethical considerations

The objective of the study was explained to the participants before data collection, and all participants included in the study gave their free and informed consent. The study protocol has been approved by the College of Natural and Computational Sciences, Addis Ababa University (Reference No: CNSDO/185/12/19).

## 3 RESULTS

### 3.1 Background characteristic of study variables

The study assessed various potential predictors for undernutrition in children aged 6–23 months at community, household, and individual levels. Even though community‐level predictors vary across the different agroecological zones, the average aggregated crop productivity across the study village in 100 kg weight (quintal) is 15.62 per hectare. Likewise, phytate to zinc and iron molar ratios were equal to 9.29 and 3.47, respectively (Table 1).

**Table 1:** Background characteristic at community, household, and individual levels.

Community‐level factors	*n* (%)	Household‐level factors	*n* (%)
*Agroecological zone*		*Wealth index*	
Midland	175 (26.5)	Low	220 (33.3)
Highland	302 (45.8)	Middle	218 (33.0)
Upper highland	183 (27.7)	High	222 (33.6)
*Phytate to zinc molar ratio (M* ± *SE)*	9.29 ± 0.07	*PSNP current membership*	
*Phytate to iron molar ratio (M* ± *SE)*	3.47 ± 0.03	Not member	495 (75.0)
*Aggregated crop yield (Quintal ha* ^*‐1*^ *) (M* ± *SE)*	15.62 ± 0.09	Member	165 (25.0)
*Food production diversity (M* ± *SE)*	3.249 ± 0.07	*Sanitation facility*	
**Individual level factors ‐ children**		Unimproved	360 (54.5)
*Age of children (months)*		Improved	300 (45.5)
6–11	226 (34.2)	*Water source for drinking*	
12–17	197 (29.8)	Unsafe	314 (47.6)
18–23	237 (35.9)	Safe	346 (52.4)
*Sex*		*Food security*	
Boys	334 (50.6)	Insecure	334 (50.5)
Girls	326 (49.4)	Secured	326 (49.4)
*Common childhood illnesses*		*Utilisation of health service*	
Present	253 (38.3)	Less frequently	144 (21.8)
Absent	407 (61.7)	More frequently	516 (78.2)
*Child dietary diversity score (CDDS)*		*Household size*	
CDDS <5	461 (69.8)	≤5	366 (55.5)
CDDS ≥5	199 (30.2)	>5	292 (44.2)
*Birth order*		*Number of children* < *5 years*	
≤2nd	328 (49.9)	1	473 (74.6)
≥3rd	332 (50.1)	≥2	161 (25.4)
*Current breastfeeding*		*Nonfarm household income*	
Yes	585 (88.6)	Households involved	146 (22.1)
No	70 (10.5)	Not involved	513 (77.7)
*Deworming in children 12*–*23 m*		*Travel time to the nearest market*	
Yes	250 (59.1)	>30 min	326 (49.4)
No	173 (40.7)	≤30 min	334 (50.6)
**Individual level factors ‐ women**			
*Education of women*	*n* (%)	*Number of pregnancies*	*n* (%)
≤Primary	421 (63.8)	≤2	314 (47.8)
≥Secondary	233 (35.3)	>2	343 (52.2)
*Nutrition knowledge of women*		*Inter‐pregnancy interval (months)*	
Below average	223 (33.8)	<24	170 (34.8)
≥Average score	437 (66.2)	≥24	319 (65.2)
*Age of women*		*Height of women (cm)*	
≤28	327 (49.5)	≤155	306 (46.6)
>28	277 (42.0)	>155	354 (53.6)
*Age of women at first pregnancy*		Women dietary diversity score	
≤19	273 (41.4)	<5	612 (92.7)
>19	253 (38.3)	≥5	48 (7.3)
*Source of nutrition‐related messages*		*Iron‐folate during last pregnancy*	
Healthcare providers	509 (77.1)	Took iron‐folate	572 (86.7)
Other sources†	150 (22.7)	Not	88 (13.3)
*Access to media* ‡		*Place of delivery*	
Have access	153 (23.2)	At health facility	577 (87.4)
No	507 (76.8)	Other	83 (12.6)

Abbreviation: ANC, antenatal care.

^†^ Other sources including friends, relatives, or neighbours.

^‡^ Women access to either print media, radio, or television.

Most households are engaged in farming, while one‐fifth of the respondents indicated that a member of their household participated in non‐farm income‐generating activities. Half of the studied households travel >30 min to reach the nearest market (reported travel time ranges between 5 min and 2 h.). With respect to the type of market visited, 46% of households visit the local village level market only and the rest have access to both the local and the next marketplace. More frequent utilisation of health service is reported from three‐fourths (78%) of the households.

One‐third of the women had at least a secondary education. Half of them are under the age of 28 years. Because most of the women had limited access to the media, more than three‐fourths of them (77%) obtained health and nutrition‐related information primarily from the healthcare providers.

During their most recent pregnancy, approximately 87% of women received iron‐folate supplementation and gave birth at a health facility. Amongst the studied women, 26% (*n* = 171) had experienced pregnancy only once, while the remaining 74% (*n* = 489) had two or more pregnancies. One‐third of the women in the latter category had an inter‐pregnancy interval of <24‐months (Table 1).

Children in the age group of 12–23 months are eligible to receive deworming drugs at regular intervals particularly in areas where soil‐transmitted helminths infestation are common (WHO, 2017). In this age group, 59% of children received deworming in the 6‐months before the survey. In the preceding 2 weeks before the survey, 38.3% of children had any one of the common childhood illnesses (fever, diarrhoea, cough, or breathing difficulty). One‐third of children (30.2%) achieved the recommended DD score of five or more food groups on the previous day of the survey (WHO/UNICEF, 2021).

### 3.2 Prevalence of undernourishment in children

**Figure 3:**
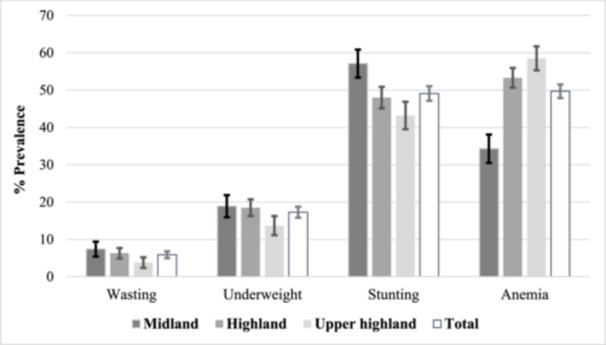
Percent prevalence of undernutrition in children 6–23 months by agroecology from South Wollo, Ethiopia, 2021.

Half of the studied children are anaemic (49.7%) and stunted (49%). Prevalence of wasting and underweight are 5.9% and 17.3%, respectively. Prevalence of stunting decreases, whereas anaemia in children increases across the agroecological gradient (see Figure 3). Midland has the highest prevalence of stunting (57%) while being lowest in anaemia (34.3%) amongst children. Based on severity, 25.9%, 22.3%, and 1.5% of the children had mild, moderate, and severe anaemia, respectively. Likewise, 28% and 21.1% of the children were moderately and severely stunted, respectively.

#### 3.2.1 Factors associated with stunting

Several potential predictors from community, household, and individual levels were related to stunting in children aged 6–23 months. Amongst individual‐level predictors, child DD score, age of the child and caregiver were significantly associated to stunting. The likelihood of stunting in children who fail to attain the minimum recommended diet diversity of five or more food groups increased by 54.3% (AOR: 1.543; 95% CI: 1.063–2.242) as compared to those qualifying for the minimum DD. Children in the 12–18 months age group had a 36% reduced risk of being stunted compared to their older counterparts. In addition, children from mothers or caregivers who were younger than the median age group of 28 years had a 58% increased risk of being stunted.

Children from households having access to non‐farm income‐generating opportunities, located closer to the market, and whose members visit health facility more frequently were significantly protected from being stunted. Households involved in non‐farm income, located closer to the market and whose members have frequently utilising health services had 42%, 38.5%, and 44% lower risks of stunting in children, respectively (Table 2).

**Table 2:** Multivariable analysis of factors associated with stunting amongst children aged 6–23 months in South Wollo, Ethiopia, 2021.

Variables	Not stunted	Stunted	COR (95% CI)	AOR (95% CI)
	*n* (%)	*n* (%)		
*Agroecological zone (AEZ)*				
Midland	75 (42.9)	100 (57.1)	1.755 (1.155, 2.668)**	1.869 (1.147, 3.043)*
Highland	157 (52.0)	145 (48.0)	1.216 (0.840, 1.759)	1.122 (0.739, 1.703)
Upper highland	104 (56.8)	79 (43.2)	1	1
*Food production diversity (M* ± *SE)*	3.194 ± 0.092	3.294 ± 0.096	1.035 (0.946, 1.132)	—
*Phy: Fe Molar Ratio (M* ± *SE)* ‡	3.37 ± 0.05	3.58 ± 0.05	1.310 (1.100, 1.561)**	1.419 (1.147, 1.756)***
*Phy: Zn Molar Ratio (M* ± *SE)* ‡	9.102 ± 0.094	9.492 ± 0.090	1.150 (1.048, 1.261)**	1.163 (1.048, 1.292)**
*Aggregated annual crop yield (Quinta**l** ha* ^*‐1*^ *) (M* ± *SE)*	15.72 ± 11.79	15.51 ± 13.69	0.999 (0.998, 1.001)	0.913 (0.845, 0.987)*
*Wealth index*				
Low	108 (49.1)	112 (50.9)	1	—
Middle	110 (50.5)	108 (49.5)	0.947 (0.651, 1.377)	—
High	118 (53.2)	104 (46.8)	0.850 (0.585, 1.234)	—
*Nonfarm household income*				
Households involved	84 (57.5)	62 (42.5)	0.707 (0.488, 1.025)	0.581 (0.383, 0.881)*
Not involved	251 (48.9)	262 (51.1)	1	1
*Travel time to the market*				
>30 min	155 (46.4)	179 (53.6)	1.442 (1.061, 1.959)*	1.615 (1.148, 2.272)**
≤30 min	181 (55.5)	145 (44.5)	1	1
*Utilisation of health service*				
Less frequent service seeking	249 (48.3)	267 (51.7)	1.637 (1.124, 2.384)*	1.558 (1.030, 2.353)*
More frequent service seeking	87 (60.4)	57 (39.6)	1	1
*Water, sanitation and hygiene practice*				
Unimproved	238 (49.6)	242 (50.4)	1.215 (0.862, 1.713)	1.247 (0.850, 1.829)
Improved	98 (54.4)	82 (45.6)	1	1
*PSNP current membership*				
Not member	254 (51.3)	241 (48.7)	1	—
Member	82 (49.7)	83 (50.3)	1.067 (0.750, 1.518)	—
*Age of women/caregiver*				
≤28 years	157 (48.0)	170 (52.0)	1	1
>28 years	150 (54.2)	127 (45.8)	0.82 (0.567, 1.078)	0.633 (0.407, 0.992)*
*Age of children*				
6–11 months	117 (51.8)	109 (48.2)	0.767 (0.523, 1.105)	1.047 (0.699, 1.568)
12–17 months	112 (56.9)	85 (43.1)	0.625 (0.427, 0.914)*	0.641 (0.432, 0.951)*
18–23 months	107 (45.1)	130 (54.9)	1	1
*Child birth order*				
2nd or lower	167 (50.9)	161 (49.1)	1	1
3rd or higher	167 (50.8)	162 (49.2)	1.010 (0.741, 1.366)	1.260 (0.807, 1.969)
*Child dietary diversity score (CDDS)*				
CDDS <5	243 (52.7)	218 (47.3)	1.271 (0.911, 1.773)	1.543 (1.063, 2.242)*
CDDS ≥5	93 (46.7)	106 (53.3)	1	1

*Note*: *, **, *** indicate statistically significant association at *p* < 0.05, *p* < 0.01 and *p* < 0.001 levels of significance, respectively.

Abbreviations: 1, reference category; AOR, adjusted odds ratio; CI, confidence interval; COR, crude odds ratio; M ± SE, mean ± standard error of the mean; PSNP, productive safety net programme.

^‡^ Values were derived from separate regression model replacing Phy:Zn and Phy:Fe molar ratios in place of AEZ because of higher collinearity amongst the variables and with the AEZ. Dashes indicate the variable was not included in the multivariable regression model.

Amongst the community‐level determinants, residence type by agroecology, aggregated annual crop productivity, phytate‐to‐iron and phytate‐to‐zinc molar ratios were found significantly associated with stunting in the multiple logistic regression analysis. Children residing in the ML were approximately twice more likely to be stunted (AOR: 1.869; 95% CI: 1.147–3.043) than those in the UHL.

A significant positive association exists between stunting and phytate to mineral molar ratios. Stunting increases by 16.3% (AOR: 1.163; 95% CI: 1.048–1.292) and 41.9% (AOR: 1.419; 95% CI: 1.149–1.756) for every unit increase in phytate to zinc and phytate to iron molar ratios, respectively. Whereas an increase in aggregated annual crop yield per hectare was associated with reduced risk of stunting. A 100 kg increase in aggregated annual crop yield per hectare reduces the likelihood of stunting by 8.7% (AOR: 0.913; 95% CI: 0.845–0.987).

#### 3.2.2 Factors associated with anaemia in children

After controlling for individual‐, household‐, and community‐level factors in a multivariable logistic regression, we found an increased odds of developing anaemia amongst boys (AOR: 1.800; 95% CI: 1.297–2.494) than girls. Likewise, children aged 6–11 months are more likely to be ananemic (AOR: 1.728; 95% CI: 1.164–2.566) as compared to those in the older age group. Children from households with unimproved water, sanitation, and hygiene (WASH) practice had a 47.7% increased risk of being anaemic (AOR: 1.477; 95% CI: 1.015–2.151) than those with improved WASH (Table 3).

**Table 3:** Factors associated with anaemia amongst children aged 6–23 months in South Wollo, Ethiopia. 2021.

Variables	Categories	Not anaemic	Anaemic	COR (95% CI)	AOR (95% CI)
		*n* (%)	*n* (%)		
*Agroecological zone*					
	Midland	115 (65.7)	60 (34.3)	1	1
	Highland	141 (46.7)	161 (53.3)	2.189 (1.489, 3.218)***	1.860 (1.145, 3.022)*
	Upper highland	76 (41.5)	107 (58.5)	2.698 (1.757, 4.143)***	2.242 (1.373, 3.660)**
*Food production diversity (M* ± *SE)*		2.92 ± 0.075	2.77 ± 0.077	0.924 (0.827, 1.032)	1.028 (0.928, 1.137)
*Per capita phytate (g day* ^*‐1*^ *) (M* ± *SE)*		2.25 ± 0.041	2.42 ± 0.036	1.407 (1.130, 1.751)**	1.136 (0.839, 1.537)
*Aggregated annual crop yield (Quintal ha* ^*‐1*^ *) (M* ± *SE)*		15.48 ± 0.138	15.76 ± 0.118	1.053 (0.986, 1.125)	0.998 (0.913, 1.091)
*Household food security status*					
	Food secured	178 (53.3)	156 (46.7)	0.785 (0.578, 1.065)	0.814 (0.578, 1.146)
	Food insecure	154 (47.2)	172 (52.8)	1	1
*Travel time to the market*					
	>30 min	155 (47.5)	171 (52.5)	1	1
	≤30 min	177 (53.0)	157 (47.0)	0.804 (0.592, 1.091)	0.741 (0.531, 1.033)
*Water, sanitation and hygiene practice*					
	Unimproved	73 (40.6)	107 (59.4)	1.718 (1.214, 2.433)**	1.477 (1.015, 2.151)*
	Improved	259 (54.0)	221 (46.0)	1	1
*Utilisation of health service*					
	Less frequent service seeking	68 (47.2)	76 (52.8)	1	1
	More frequent service seeking	264 (51.2)	252 (48.8)	0.854 (0.590, 1.236)	0.970 (0.654, 1.441)
*Women nutrition knowledge*					
	Average or above	107 (48.0)	116 (52.0)	0.869 (0.629, 1.200)	0.907 (0.640, 1.285)
	Below average	225 (51.5)	212 (48.5)	1	1
*Role of women in HH decision making*					
	Involved	170 (51.7)	159 (48.3)	0.897 (0.661, 1.217)	0.957 (0.690, 1.328)
	Not involved	162 (48.9)	169 (51.1)	1	1
*Gender of a child*					
	Boy	148 (44.3)	186 (55.7)	1.629 (1.198, 2.212)**	1.800 (1.297, 2.494)***
	Girl	184 (56.4)	142 (43.6)	1	1
*Age group of children*					
	6‐11 months	88 (38.9)	138 (61.1)	1.971 (1.361, 2.855)***	1.728 (1.164, 2.566)**
	12‐17 months	112 (56.9)	85 (43.1)	0.954 (0.652, 1.397)	0.930 (0.625, 1.384)
	18‐23 months	132 (55.7)	105 (44.3)	1	1
*Child dietary diversity score (CDDS)*					
	CDDS < 5	224 (48.6)	51.4 (51.4)	1.256 (0.900, 1.753)	1.352 (0.938, 1.948)
	CDDS ≥ 5	108 (54.3)	91(45.7)	1	1
*Common childhood illnesses*					
	Present	121 (47.8)	132 (52.2)	1	1
	Absent	207 (50.9)	200 (49.1)	0.886 (0.647, 1.212)	0.892 (0.635, 1.254)

*Note*: *, **, *** indicate statistically significant association at *p* < 0.05, *p* < 0.01, and *p* < 0.001 levels of significance, respectively.

Abbreviations: 1, reference category; AOR, adjusted odds ratio; CI, confidence Interval; COR, crude odds ratio; HH, household; M ± SE, mean ± standard error of the mean; WASH, water, sanitation and hygiene.

Children are more likely to be anaemic as the agroecological gradient increases. Children in HL and UHL are 1.86 and 2.24 times more likely to be anaemic than those in ML. Other community‐level determinants were not associated with anaemia in the multivariate model, whereas in the binary logistic regression, a unit increase in per capita phytate produced (g day^−^^1^) at the community level was associated with a 40.7% increased risk of children being anaemic (COR: 1.407; 95% CI: 1.130–1.751).

## 4 DISCUSSION

The present study aimed to assess the prevalence and determinants of stunting and anaemia in children aged 6–23 months from rural Ethiopia. Half of the studied children are found to be stunted (49%) and anaemic (49.7%), making both conditions severe public health problems according to de Onis et al. (2018) and WHO (2024). The determinants were evaluated at community‐, household‐, and individual‐levels. Occurrence of stunting was significantly correlated to differences in agroecology, aggregated crop yield, and phytate‐to‐mineral molar ratios at community level. Amongst household level predictors, nonfarm income, travel time to the market, and utilisation of health facility were associated with stunting. Child DD, age of the child and mother were significantly associated individual level factors with stunting, whereas anaemia in children was associated with differences in agroecology, household WASH, gender and age of children.

### 4.1 Stunting

Prevalence of stunting shows a significant variation across the age groups of children (see Supporting Information S1: Figure S1). Stunting becomes prevalent between the age of 9 and 11 months. This is presumably due to a combination of inadequate or delayed introduction of complementary feeding, and hence providing breastmilk alone, which is insufficient to deliver all the required nutrients after 6 months. In the subsequent age group, 12–17 months, children had the lowest prevalence of stunting than in the other age groups, while the prevalence peaks towards the near age 2 (18–23 months). This could be partly related to reduced childcare with early transition from complementary feeding to family food. This pattern of stunting prevalence by age has been similarly observed in the 2019 mini‐demographic and health survey of Ethiopia (EPHI and ICF, 2019).

The present study shows that stunting appears to be common throughout the transitional periods, both from exclusive breastfeeding to the introduction of complementary food (CF) and CF to family food (see Supporting Information S1: Figure S1). The reason is that the transitions did not follow the recommendation of being age appropriate, safe, smooth enough with adequate child feeding and continued care. Studies also confirm that inappropriate complementary feeding practice is the most common factor for stunting (Hijra et al., 2016; Tello et al., 2022).

In agreement to the present finding, previous studies have also showed that poor child health outcomes and growth restrictions occur before or around age 2 (Karlsson et al., 2022; Karlsson et al., 2023). Karlsson et al. (2023) evaluated patterns of child stunting by age from numerous low‐ and middle‐income countries. Even though they observed a higher prevalence of stunting at all ages in regions with low socioeconomic status, a sharper positive age gradient was seen immediately before the age of 2 in most contexts. This is presumably due to the chronic accumulation of adverse exposures to undernutrition and infections.

Further analysis on disaggregated data by agroecological zones have showed that the prevalence of stunting was quite higher in ML than in other AEZs. The risk of children being stunted in the ML is approximately two‐fold larger than in the UHL. Previous studies present mixed findings. In line with this finding, Randell et al. (2020) showed that either higher temperature or lower rainfall during early life (*in utero*) was associated with an increased risk of stunting in Ethiopia. In contrary, Baye and Hirvonen (2020) using data from numerous low‐ and middle‐income countries showed that increasing altitude was associated with increased risk of stunting. The difference could be related to variation in other contextual factors affecting linear growth and yet to be validated with longitudinal studies.

A previous study conducted in the study area by Guja et al. (2024) showed an increasing pattern of household‐level dairy production and consumption amongst children across the agroecological gradient. Such increase in milk consumption over time are robustly associated with huge reductions in child stunting (Haile & Headey, 2023; Herber et al., 2020). Milk contains calcium and insulin‐like growth factor‐1, both of which are important for linear growth of children in addition to providing energy and protein (Hoppe et al., 2006).

The pattern of stunting prevalence varies greatly with age in the three AEZs (Supporting Information S1: Figure S2). In ML, the prevalence of stunting in children is consistently higher across 9–23 months of age. Following the introduction of CF at 6–8 months, a sharp rise in stunting prevalence was observed in children aged 9–11 months. This could be related to a lower diet diversity score that is superimposed by unhealthy environment including unsafe water source that perpetuates childhood illnesses when compared to the other AEZs (Supporting Information S1: Table S1; Supporting Information S1: Figure S3). For instance, the proportion of households with unsafe water source were 62.3% and 33.3% in ML and UHL, respectively. Likewise, in 2 weeks before the survey, twice as many children in ML (35.4%) as compared those in UHL (18.0%) had a febrile illness. Fever most often occurs in response to infectious disease. Frequent infections in turn contribute to childhood stunting through reduced intake of food, malabsorption, loss, and increased requirements of nutrients (Walson & Berkley, 2018). Additionally, common childhood illnesses are observed to suppress growth hormone level in infants (Jones et al., 2015).

Unlike in the other AEZs, at early age (6–9 months), the prevalence of stunting is considerably higher in HL (57.1%), as compared to ML (42.6%) and UHL (35.6%). Even though the age of introducing any CF is comparable across all the AEZs, early introduction of nutrient dense food groups including ASF, fruits, and vegetables are practiced less in HL. The mean age of introducing these food groups is 6.7 months in UHL, while it is beyond 7.6 months in HL (Supporting Information S1: Table S2).

Furthermore, amongst women who gave birth more than once, 59.3% of them had short inter‐pregnancy interval (<24 months) in the HL. While the proportions were relatively smaller in the other AEZs, 38.2% in ML and 23.8% in UHL (Chi‐square test, *p* = 0.01). A recent meta‐analysis by Ntambara et al. (2023) showed that children from mothers with longer inter‐pregnancy interval ( >24 months) had a 40% lower risk of stunting. Owing to short inter‐pregnancy interval, mother's nutrient reserves become depleted, and this increases the risk of intrauterine growth retardation that negatively impacts the infant's nutrient stores at birth and nutrient supply through breastmilk (Chungkham et al., 2020). The present study suggests further follow‐up study that will examine environmental covariates, the health and nutrition status of women during pregnancy, evaluating birth weight and birth length of newborns across the different agroecology could explain variation in the prevalence of stunting that has been observed in infants before or at the onset of complementary feeding age.

Children from households having less frequent health service utilisation had a 55.8% increased risk of being stunted. In line with this finding, Kahssay et al. (2020) observed that children from mothers who had no health facility visit during pregnancy were approximately three times more likely to be stunted than those mothers that had the follow up. This is partly because, as observed in the present study, most household members in rural areas obtain health and nutrition‐related information from the healthcare providers.

Children from households whose members are engaged in nonfarm income‐generating activities were 42% less likely to be stunted than children from households whose only source of income is farming. According to numerous studies (Guja et al., 2024; Liu et al., 2023; Sibhatu et al., 2015), nonfarm income increases DD through the market and promote access to and utilisation of health services (Mishra & Chang, 2012). This is particularly presumed to be correct in farming households that are relying on smaller farmlands.

We observed that food production diversity (FPD) was not associated with the occurrence of stunting. Whereas children from households located in short travel time to the market had 61.5% reduced risk of being stunted. This suggests that the role of market is higher in reducing the risk of stunting than FPD. This could be achieved through increased child DD. Since this study additionally showed that children who fail to achieve the minimum DD score of five or more food groups had 54.3% increased risk of being stunted. When data was further disaggregated by AEZ, however, showed that child DD consistently scored higher in HL (Supporting Information S1: Figure S3), being an AEZ that is known for producing more diverse foods than the other two (Guja et al., 2024). This shows that in settings where FPD is considerably high, the risk of stunting could be reduced provided that FPD translates into child DD.

Aggregated crop productivity at the community level appears to be associated significantly with stunting. A 10% increase in the mean aggregated productivity reduces the likelihood occurrence of stunting by 13.6%. In line with this finding, a study from Nigeria by Amare et al. (2020) showed that a 10% increase in agricultural productivity reduced stunting by 2%. Likewise, in a study analysing progress on national‐level stunting reduction in Ethiopia, Tasic et al. (2020) indicated that increasing yield was amongst the most rewarding approaches for the reduction of stunting prevalence achieved in the country between 2000 and 2016. In contrast, the World Bank report (2013) argued that agriculture production through increased yields of staple crops improves food availability and household incomes but has had limited success in improving nutritional status. This shows heterogeneity in the impact of agricultural productivity on child nutritional outcomes. Increased productivity is presumed to play a significant role in stunting reduction in places where aggregated food production is insufficient to sustain year‐round food availability. Production side deficit in gross energy and essential nutrients have already been indicated in the study area (Guja et al., 2023). In line with this, Tessema et al. (2019) showed that linear growth of children was positively associated with gross energy and protein intakes in Ethiopia. Furthermore, Amare et al. (2020) showed that agricultural productivity has a higher impact on stunting in children for households that have better access to the market and higher level of parental education. This highlights that adequacy of food production in a given setting, access to the market, and nutrition knowledge are amongst the factors determining how agriculture productivity and the generated income are invested to promote diet and improve nutritional status.

Lower bio‐availability of essential nutrients are observed to significantly increase occurrence of stunting. This study showed that a unit increase in phytate‐to‐zinc and phytate‐to‐iron molar ratios increased the risk of stunting by 16% and 42%, respectively. This is related to the significant role of these mineral micronutrients in promoting linear growth. Stunting is strongly associated with iron and zinc deficiency, both of which are still common in low‐income countries (Black et al., 2008). A study from Uganda showed that multiple micronutrient deficiencies were found in stunted children, with almost half of the stunted children had iron deficiency (Mutumba et al., 2023). Similarly, the role of zinc in promoting linear growth is well documented (Imdad & Bhutta, 2011; Imdad et al., 2023; Wastney et al., 2018). A randomised controlled trial amongst stunted infants in rural Ethiopia showed zinc supplementation significantly increased linear growth of infants, reduced susceptibility to infection, and improved appetite (Umeta et al., 2000). Therefore, interventions addressing stunting should target co‐existing micronutrient deficiencies through various approaches including reducing concentrations of antinutrients.

### 4.2 Anaemia in children

Half of the studied children are anaemic. According to the 2016 Ethiopian demographic and health survey, 57% of children under the age of 5 were anaemic (CSA and ICF, 2016). While children aged 6–23 months had the highest prevalence (72%). Similar disparities in anaemia prevalence were observed in children under 5 (Belachew & Tewabe, 2020) and amongst infants 6–23 months (Azmeraw et al., 2023), respectively, reporting pooled prevalence of 44.8% and 57.76% in Ethiopia. This indicates the condition is still a severe public health problem in the country (WHO, 2024) and younger children are predominantly affected. The use of different Hb cut‐offs to define anaemia amongst children aged 6–23 months could be the reason for the discrepancy in prevalence. Previous studies used <11 g dL^−^^1^ (WHO, 2011), while the present study used <10.5 g dL^−^^1^ (WHO, 2024).

Amongst community‐level determinants, resident type by agroecology is significantly associated with occurrence of anaemia. Children from the midland agroecological zone were more protected from being anaemic. This could be partly related to a decreased consumption of cow milk. In this study, a dose–response relationship between cow milk consumption and prevalence of anaemia was observed across the agroecological gradient (Supporting Information S1: Figure S4). Because of huge intra‐household variation in animal source foods consumption, children are favoured to consume more milk and egg than women (Guja et al., 2024). Several studies indicated the association between poultry ownership or egg consumption and reduced occurrence of anaemia in children (Omer et al., 2023; Zerfu et al., 2023). However, as in most other smallholder farming households (Bonis‐Profumo et al., 2021; Bundala et al., 2020; Lambrecht et al., 2021), a considerable proportion of eggs produced at the studied households are sold in the local market due to the competing interest of household income (Guja et al., 2024). However, the authors indicated that consumption of cow's milk go along with household production.

A similar trend of anaemia prevalence in children has been observed across Ethiopian regions with varying proportions of household cow milk consumption (CSA and ICF, 2016; Jateno et al., 2023). A consistently higher prevalence of anaemia in children was observed in Afar and Somali regions where cow milk is predominantly consumed (Supporting Information S1: Figure S5).

Infants have a high demand for iron because of their rapid growth. In such circumstances, providing cow milk would predispose them to iron deficiency (Griebler et al., 2016; Ziegler, 2007). This is mainly attributed to the low iron content, high contents of milk protein called casein, and calcium in cow milk. Casein in milk chelates iron by binding with its phosphoserine residues preventing the release of iron in a free form. This consequently hinders intestinal absorption of iron by infants (Kibangou et al., 2005). Additionally, Roughead et al. (2005) showed that calcium inhibits both haem and nonheme iron absorption in a dose‐dependent manner. As a result, in places where cow milk is regularly provided as part of complementary feeding, the occurrence of anaemia in infants/children increases (Abdurahman & Gashu, 2021; Gupta, 2017; Hadler, 2004).

This study showed that younger children (6–11 months) had a higher prevalence of anaemia than their older counterparts. Similar findings were reported from several studies (Azmeraw et al., 2023; Gebreweld et al., 2019). This could be related to the delayed introduction of nutrient dense CFs and increased susceptibility to infection (Armitage & Moretti, 2019; Gao et al., 2013; Gebremedhin et al., 2017). In addition, breast milk, unfortified cow milk, and locally prepared cereal‐based CFs are poor iron source—all in aggregate at early infancy predispose to anaemia (Gibson et al., 2010; Gupta, 2017). Owing to this, it is advised to minimise or avoid regular consumption of cow's milk during the first 12 months of life to prevent iron deficiency anaemia (Kazal LA, 2002; Koletzko et al., 2019).

Again, we observed a significant sex difference in the prevalence of anaemia. With increased odds of developing anaemia amongst boys than girls. Studies from different region of the world have similarly showed this association (Keokenchanh et al., 2021; Moschovis et al., 2018; Onyeneho et al., 2019). This could be explained by the physiological variations caused by faster growth velocity in boys than girls (Gao et al., 2013; Zetterströ, 2004), which necessitates a higher demand for iron, particularly during the early years of life.

Per capital phytate (g day^−^^1^) and aggregate annual crop yield were not associated with anaemia in the multivariable model. While in the bivariate analysis, significant associations were observed. A unit increase in per capita phytate production at community‐level increases the risk of anaemia occurrence in children by 40.7% (COR: 1.407; 95% CI: 1.130–1.751). Unlike the gross energy supply, increased productivity of staples may not ensure equivalent production of essential nutrients (Geyik et al., 2020; Guja et al., 2023).

As the only household‐level variable found to be associated with anaemia, a child from household with unimproved WASH has a 47.7% increased risk of being anaemic than those with improved WASH. In line with this, Gizaw and Worku (2019) showed that a combined WASH has a paramount benefit to improve nutritional status in younger children. This is achieved through reduced incidence of diarrhoeal diseases, environmental enteric dysfunction, and parasitic infections (Team, 2015; Yu et al., 2020).

### 4.3 Strengths and limitations

This study has several strengths. The study assessed the two most prevalent forms of child undernutrition with slower rate of reduction overtime and whose contributing factors were not fully understood. The study comprehensively evaluated factors nested at community, household, and individual levels. For the first time, child undernutrition was related to production of antinutrients at community‐level. In addition, landscape agroecology and food production‐related variables were considered. The cross‐sectional nature of this study limits us from making causal inference of the relationship.

## 5 CONCLUSION

In areas presenting huge contextual variations, disregarding predictors nested at community‐level may affect inferences made about nutrition outcomes in children. Prevalence of stunting and anaemia in children is unacceptably high and varies significantly across the agroecological zones. Increased molar ratios of antinutrient to iron and zinc are associated with increased risk of stunting. Rise in aggregated community‐level crop productivity greatly reduces the risk of stunting in children than anaemia. At the household level, use of health facilities, access to the market, and involvement in nonfarm income reduce the risk of stunting, while improved WASH reduces occurrence of anaemia. Stunting is more common in children aged 18–23 months, but anaemia is more common in children aged 6–11 months. Therefore, the influence of these contributing factors should be considered to tailor strategies for reducing undernutrition in children 6–23 month of age in rural Ethiopia. Interventions to reduce child undernutrition should go beyond the administrative boundaries into targeting agroecological differences.

## AUTHOR CONTRIBUTIONS

All authors contributed to the study conception and design. Habtamu Guja performed material preparation, data collection, data analysis and wrote the first draft. Mariana Belgiu, Kaleab Baye, and Alfred Stein guided the research and reviewed the manuscript. All authors read and approved the final manuscript.

## CONFLICT OF INTEREST STATEMENT

The authors declare no conflict of interest.

## Supporting information

Supporting information.

## Data Availability

All data generated or analysed during this study are included in this article and its supporting information. If more data are needed for specific purposes, these are available from the corresponding author upon reasonable request.
