# Supplementary material for: Prevalence and determinants of stunting and anaemia in children aged 6–23 months: A multilevel analysis from rural Ethiopia
Source: Matern Child Nutr. 2024 Oct 8;21(1):e13736. doi: 10.1111/mcn.13736 (PMC11650036; doi:10.1111/mcn.13736)
Supplement: Supplementary file 1 — Supporting information. [file MCN-21-e13736-s001.docx]

**Prevalence and determinants of stunting and anemia in children aged 6-23 months: a multilevel analysis from rural Ethiopia.**

*Maternal & Child Nutrition*

**Supporting Information**

**FIGURE S1** Percent prevalence of stunting by age group from the present study and the 2019 Ethiopian demographic and health survey (EPHI & ICF, 2019).


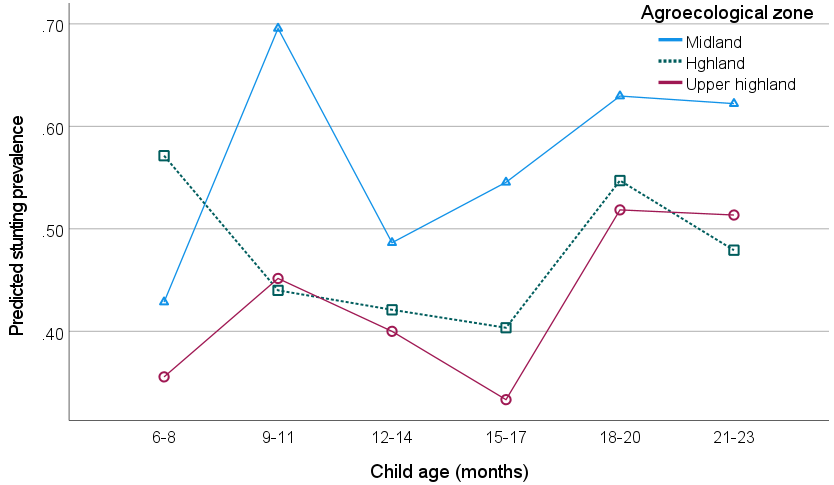


**FIGURE S2** Predicted stunting prevalence by child age group across the three agroecological zones from rural South Wollo, Ethiopia.

| **TABLE S1** Household WASH and illness among children | | | | |
| --- | --- | --- | --- | --- |
|  | Midland | Highland | Upper highland | p-value |
| Sanitation facility (unimproved) | 116(66.3) | 171(56.6) | 73(39.9) | <0.001 |
| Water source (unsafe) | 109(62.3) | 144(47.7) | 61(33.3) | <0.001 |
| Hygiene practice (poor) | 116(66.3) | 171(56.6) | 73(39.9) | <0.001 |
| Illness among children | 79(45.1) | 108(35.8) | 66(36.1) | 0.097 |
| WASH: Water, Sanitation and Hygiene.  Values were presented as n (%) and p-value resulting from Chi-square test.  When a child is presented with any common childhood illness (fever, diarrhea, cough or breathing difficulty) in the preceding 2 weeks prior to the survey. The WASH indicators categorized as unimproved according to the Joint Monitoring Program (WHO/UNICEF, 2017; EDHS, 2016). | | | | |


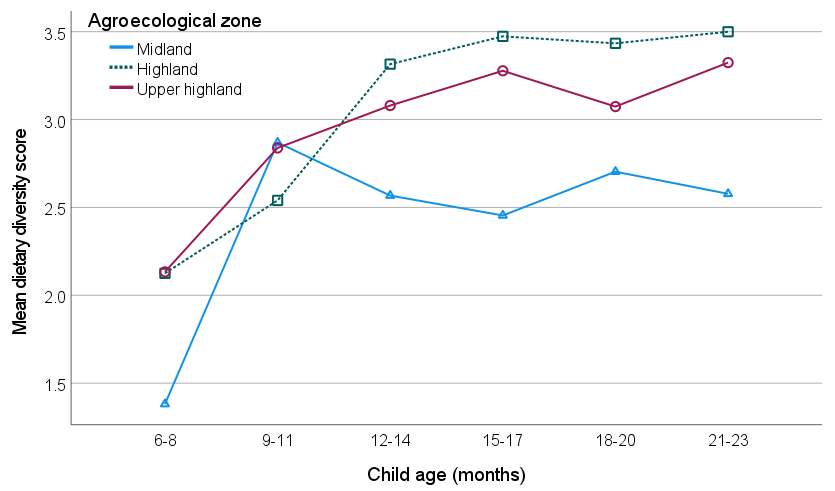


**FIGURE S3** Mean child dietary diversity score by age group across the three agroecological zones. Child dietary diversity is determined out of seven food groups (WHO, 2008).

| **TABLE S2** Age of child and complementary feeding practice by agroecological class | | | | |
| --- | --- | --- | --- | --- |
|  | Midland | Highland | Upper highland | p-value |
| Age of introducing any CF | 5.88±1.21 ^a^ | 5.96±0.84 ^a^ | 6.02±0.52 ^a^ | 0.307 |
| Age of introducing ASF | 7.38±2.09 ^b^ | 7.65±2.37 ^b^ | 6.74±1.54 ^a^ | <0.001 |
| Age of introducing fruits | 7.33±2.20 ^b^ | 7.65±2.13 ^b^ | 6.63±1.25 ^a^ | <0.001 |
| Age of introducing vegetables | 7.17±2.17 ^a^ | 7.69±2.49 ^b^ | 6.76±1.57 ^a^ | <0.001 |
| ***‡*** Age of child start family food | |  |  |  |
| 9-11m | 23(13.5) | 67(22.3) | 22(12.2) |  |
| At 12m | 53(31.0) | 109(36.3) | 64(35.6) | 0.011 |
| 13-23m | 48(28.1) | 54(18.0) | 48(26.7) |  |
| ≥ 24m | 47(27.5) | 70(23.3) | 46(25.6) |  |
| CF Complementary food. ASF Animal source foods.  Values were presented mean± SD, p-values derived from one-way ANOVA.  Values followed by different superscript letters across a row are significantly different.  ***‡*** Values were presented as n (%) and p-value resulting from Chi-square test. | | | | |

**FIGURE S4** Household consumption of cow milk and prevalence of anemia in children by agroecology. The consumption of cow milk was obtained from a reference period of 7 days.

**FIGURE S5** Proportion of households consume cow milk and prevalence of anemia in children by region in Ethiopia. SNNP: Southern nations, nationalities and peoples’ region. Source: secondary data analysis from data based on CSA & ICF, 2016; Jateno et al., 2023.
